# Education correlate with hippocampus volumes in African American males but not in females

**DOI:** 10.1002/alz.093148

**Published:** 2025-01-09

**Authors:** Darlingtina Esiaka, Erin L. Abner, David K Powell, Gregory A Jicha, Yang Jiang

**Affiliations:** ^1^ University of Kentucky College of Medicine, Lexington, KY USA; ^2^ Department of Epidemiology, University of Kentucky, Lexington, KY USA; ^3^ Sanders‐Brown Center on Aging, University of Kentucky, Lexington, KY USA; ^4^ University of Kentucky, Lexington, KY USA; ^5^ University of Kentucky Sanders‐Brown Center on Aging, Lexington, KY USA

## Abstract

**Background:**

Prior research shows that education is a protective factor against cognitive decline. Education can contribute to cognitive reserve and influence the formation and efficiency of neural networks in the brain. Brain volume, particularly changes in specific brain regions, such as the hippocampus, amygdala, and precuneus, plays a significant role in cognitive decline. Reduced brain volumes are associated with impairments in episodic memory and decline in higher‐order cognitive processes, a hallmark and predictive marker seen in conditions like Alzheimer’s disease. Research also suggests that brain volume measures serve as predictive markers for cognitive decline and may aid in the early detection of neurodegenerative diseases. The current study explored sex differences in the association between education and brain volumes in older Black Americans.

**Method:**

Data from 38 African American participants (M_age_ = 72.81, SD = 8.43; 26 females) were derived from the University of Kentucky Alzheimer’s Disease Research Center (UK‐ADRC) cohort, and their years of education ranged from 10‐21 years (M = 15.14, SD = 2.99). The participants completed a series of tests, including a review of health history, physical and neurological exams, neuropsychological testing, and MRI scans in accordance with procedures at UK‐ADRC.

**Result:**

We found that, in African American males, higher years of education were significantly associated with increased volume in the left hippocampus (r = .801, p <.01) and right hippocampus (r = .834, p <.01). Results also showed a trending negative association between higher levels of education and right amygdala (r = ‐.612, p = .06) in African American males. We observed no association between education and brain volumes in African American females.

**Conclusion:**

Our findings suggest that contextual differences may interact with educational attainment to impact brain development in males versus females. Also, educational experiences may have varying effects on neuroplasticity, and brain volume changes over the lifespan, influencing cognitive aging differently in males and females. Studying how education interacts with brain volumes in males versus females can contribute to a more nuanced understanding of the relationship between education and cognitive decline. This knowledge can inform preventive measures and interventions sensitive to the unique needs and vulnerabilities of both males and females.

